# Targeting Antibiotic Resistance Genes Is a Better Approach to Block Acquisition of Antibiotic Resistance Than Blocking Conjugal Transfer by Recipient Cells: A Genome-Wide Screening in *Escherichia coli*

**DOI:** 10.3389/fmicb.2019.02939

**Published:** 2020-01-08

**Authors:** Kazuki Moriguchi, Fatin Iffah Rasyiqah Mohamad Zoolkefli, Masanobu Abe, Kazuya Kiyokawa, Shinji Yamamoto, Katsunori Suzuki

**Affiliations:** ^1^Program of Basic Biology, Graduate School of Integrated Sciences for Life, Hiroshima University, Higashihiroshima, Japan; ^2^Department of Biological Science, Graduate School of Science, Hiroshima University, Higashihiroshima, Japan; ^3^Division for Health Service Promotion, University of Tokyo, Tokyo, Japan

**Keywords:** IncP1α-type plasmid, spread of antibiotic resistance genes, broad host range plasmid, conjugal transfer, genome-wide screening, recipient mutants

## Abstract

The conjugal transfer is a major driving force in the spread of antibiotic resistance genes. Nevertheless, an effective approach has not yet been developed to target conjugal transfer to prevent the acquisition of antibiotic resistance by this mechanism. This study aimed to identify potential targets for plasmid transfer blockade by isolating mutants defective in the completion of the acquisition of antibiotic resistance via conjugal transfer. We performed genome-wide screening by combining an IncP1α-type broad host range plasmid conjugation system with a comprehensive collection of *Escherichia coli* gene knockout mutants (Keio collection; 3884 mutants). We followed a six-step screening procedure to identify the mutants showing conjugation deficiency precisely. No mutants defective in the conjugal transfer were isolated, strongly suggesting that *E. coli* cannot escape from being a recipient organism for P1α plasmid transfer. However, several mutants with low viability were identified, as well as mutants defective in establishing resistance to chloramphenicol, which was used for transconjugant selection. These results suggest that developing drugs capable of inhibiting the establishment of antibiotic resistance is a better approach than attempting to prevent the conjugal transfer to block the spread of antibiotic resistance genes. Our screening system based on the IncP1α-type plasmid transfer can be extended to isolation of target genes for other drugs. This study could be the foundation for further research to understand its underlying molecular mechanism through functional analysis of the identified genes.

## Introduction

The threat of antibiotic resistance is an ongoing source of serious concern for human health (Asaduzzaman, 2018; Zumla et al., 2018). Antibiotic overuse, in both medical treatments and farming, has caused a strong selective pressure allowing the emergence of drug-resistant pathogens. Insufficient sewage treatment and the progress of globalization have also aggravated the spread of drug-resistant bacteria worldwide (Bengtsson-Palme et al., 2014; Brown and Wright, 2016; Thanner et al., 2016). Although efforts to develop new antibiotics are strenuously being pursued, there may be a possibility that a multi-drug resistant pathogen could emerge that is resistant to all existing antibiotics. Furthermore, the emergence of a resistance gene against colistin, which is used as a last resort antibiotic, has been reported in recent years (Liu et al., 2016; McGann et al., 2016; Davies and Walsh, 2018).

Antibiotic resistance that arises in a single bacterium can easily spread to other bacteria via the conjugal transfer of the resistance gene, which is a major driving force for the transfer of antibiotic resistance genes. These genes are usually transferred through vectors such as plasmids or integrative conjugative elements (ICE) (Thomas and Nielsen, 2005; Amábile-Cuevas, 2013). Various methods have been tested to block conjugal transfer to control the antibiotic resistance crisis. Three groups of conjugation factors have been proposed: those encoded on the donor chromosome, those encoded on plasmids, and those encoded on the recipient chromosome. If these conjugation-related factors are evolutionarily well-conserved and can be characterized, and if effective inhibitory drugs capable of targeting these factors could be developed, the risk of multi-antibiotic resistance could be decreased.

Several factors in the first group, for example, Sfr, have been reported to be involved in the transfer of F and F-like plasmids (Beutin and Achtman, 1979; Kato and Katayama, 2001; Starcic et al., 2003; Williams and Schildbach, 2007). Recently, a non-peer reviewed article reported the existence of more than 50 novel candidates identified by a genome-wide screening of a comprehensive collection of knockout *Escherichia coli* mutants and the F plasmid, although these have not been fully confirmed (Alalam et al., 2018). In the case of the second group, although the factors such as Tra and Trb and their functions are well characterized, the discovery of effective inhibitory molecules is only now underway and promising results have been reported (Shaffer et al., 2016). For the third group of factors, a genome-wide screening was performed in *E. coli* using conjugation of the IncW plasmid R388; however, no factors essential for conjugation were isolated, except for enzymes in the lipopolysaccharide (LPS) synthesis pathway, although these had only modest effects (6–32% of wild type). Based on this, the authors of this latter study concluded that recipient bacterial cells cannot avoid being used as recipients in bacterial conjugation (Pérez-Mendoza and de la Cruz, 2009). However, the universality of this conclusion with respect to conjugal transfer, and alternative methods, which can protect against the emergence of resistance in plasmid-incorporated cells, have not been well studied, despite reports in the 1970s showing that defects in LPS and the outer membrane protein OmpA cause a conjugation deficiency for the F plasmid (Watanabe et al., 1970; Skurray et al., 1974).

In order to identify drug targets that would be effective in blocking the emergence of antibiotic resistance by conjugal transfer, we aimed to isolate recipient mutants via conjugal transfer, by combining the IncP1α plasmid transfer system (derived from RP4) with a comprehensive collection of *E. coli* gene knockout mutants (the Keio collection; 3884 mutants). IncP plasmids are a group of broad host range, low copy number plasmids found in *Enterobacteriaceae* as well as *Pseudomonas* spp. They have been mainly isolated from *E. coli* and *Klebsiella pneumoniae* in human samples (Rozwandowicz et al., 2018). RP4 (also known as RK2 or RP1) was found in the clinically isolated strain S8 of *Pseudomonas aeruginosa* (Datta et al., 1971; Saunders and Grinsted, 1972). This plasmid has a broad host range among gram-negative bacteria and has broad transfer range even in eukaryotes and archaea (Heinemann and Sprague, 1989; Dodsworth et al., 2010; Moriguchi et al., 2013a, b; Karas et al., 2015). This characteristic of the RP4 plasmid strongly suggests that recipient organisms have little choice over whether or not conjugation occurs.

## Materials and Methods

### Bacterial Strains and Plasmids

The *E. coli* strains and plasmids used are listed in Table 1. The complete set of *E. coli* deletion clones (Keio collection) was provided by the National BioResource Project (NBRP) of Ministry of Education, Culture, Sports, Science and Technology (MEXT), Japan. Although the total number of strains provided was 3909, 25 strains were removed from screening because precise gene disruption had not occurred in these strains (Yamamoto et al., 2009). BW25113 (pBBR122Δ*Cm*^R^) was used as the control strain.

**TABLE 1:** *E. coli* strains and plasmids used in this study.

**Strains and plasmids**	**Relevant characteristics**	**Source or reference**
**Strains**		
HB101	*F^–^ hsdS20(r^–^_B_ m^–^_B_) recA13 ara-14 proA2 lacY1 galK2 rpsL20 xyl-5 mtl-1 supE44 λ^–^ leu thi*	NBRP Japan
S17-1 λ*pir*	*F^–^ RP4-2(Km*^R^*::Tn7,Tc*^R^*::Mu-1) pro-82* λ*pir recA1 endA1 thiE1 hsdR17 creC510*	NBRP Japan
BW25113	*F*^–^Δ*(araD-araB)567*Δ*lacZ4787(::rrnB-3) λ^–^ rph-1*Δ*(rhaD-rhaB)568 hsdR514*	NBRP Japan
Keio collection	An in-frame single-gene knockout mutant collection derived from BW25113, *Km*^R^	NBRP Japan
**Plasmids**		
pBBR122Δ*Cm*^R^	Derivative of a commercially provided plasmid vector pBBR122, *Rep*^*pBBR*^′(non-transmissible) *Km*^R^	This study
pRS316::*oriT*^*P*^	*URA3 CEN6/ARSH4 ori*-pMB1 *Ap*^R^ *oriT*^RP4^	Moriguchi et al., 2013b
pRH220	*tra*^RP4^ *trb^RP4^ oriT*^RP4^ *ori*-pSC101 *Cm*^R^	^∗^AB526840
pJP5603Δ*Km*^R^(*::Gm^R^ Cm*^R^ *leuB*-*D*)	*ori*-R6K *oriT*^RP4^ *lacZ*αΔ*Km*^R^(*::Gm^R^ Cm*^R^ *leuB*-*D*)	This study
RP4Δ*Km*^R^(*::Gm^R^*)	*tra trb oriT oriV Ap*^R^ *Tc*^R^ Δ*Km*^R^(*::Gm^R^*)	This study

*^∗^DDBJ/EMBL/GenBank accession number.*

### Donor and Recipient Cell Culture

LB Lennox (LB: 1% tryptone, 0.5% yeast extract, 0.5% NaCl, 1.5% agar if necessary) medium was routinely used for *E. coli* culture. The antibiotics chloramphenicol (Cam; 30 μg/mL), ampicillin (Amp; 50 μg/mL), kanamycin (Kan; 50 μg/mL), tetracycline (Tet; 7.5 μg/mL), and gentamycin (Gen; 30 μg/mL) were supplemented as necessary.

For the donor culture, in the primary and secondary screenings, HB101 (pRH220 pRS316::*oriT*^*P*^) was cultured with medium supplemented with Cam and Amp at 5 mL scale using glass tubes for 16 to 18 h at 37°C following inoculation from a solid culture. For the third and fourth screenings, S17-1 λ*pir* [pJP5603Δ*Km*^R^(*::Gm^R^ Cm^R^ leuB*-*D*)] and HB101 (RP4Δ*Km^R^::Gm^R^*) were cultured in a similar manner, and the cultures were supplemented with Cam and Amp, respectively. For the fifth and sixth screenings (i.e., the final confirmation steps), HB101 (RP4Δ*Km^R^::Gm^R^*) and HB101 (pRH220) were cultured in media supplemented with Amp and Cam, respectively.

For the recipient culture, the Keio mutants were inoculated from 96-well frozen stock plates and cultured at 100 μL scale using 96-well flat-bottom plates at 37°C for 22–24 h for primary screening, and for 20–22 h for the second to fourth screenings. In the fifth and sixth screenings, the culture scale was increased up to 600 μL, and cultured at 37°C for 20–22 h using 5 mL disposable plastic tubes.

### Conjugation Reaction

For the primary screening, to provide sufficient statistical power in our calculation of the median value for the conjugation efficiency, we defined two 96-well Keio mutant strain plates (comprising 48 plates in total) as one conjugation experiment set, and 50 μL each of the donor and recipient cell cultures were mixed and incubated for 24 h at 28°C (conjugation reaction). After the conjugation reaction was complete, the reaction mixtures were well suspended and then 10 μL of each mixture was diluted with 90 μL of TNB [80 mM Tris–HCl (pH 7.5) and 0.05% NaCl]. Subsequently, 10 μL of each diluted mixture was inoculated into 90 μL of fresh medium containing Amp, Cam, and Kan in 96-well flat-bottom plates. After incubation for 24 h at 37°C using a plate shaker, the growth of transconjugants was assessed by measuring the turbidity at a wavelength of 600 nm, using a microtiter-plate reader MTP-310 (CORONA, Ibaraki, Japan).

For the second to fourth screening steps, donor cells were collected by centrifugation and re-suspended in TNB at an OD 660 nm value of 1.8. Recipient cultures in a 96-well flat-bottom plate were then moved to a 96-well v-bottom plate, and the cells were collected by centrifugation and re-suspended in 100 μL of TNB. Following this, 50 μL each of the donor and recipient cells were mixed and incubated for 1 h at 28°C. After the conjugation reaction, the mixtures were well suspended, and then diluted with TNB to the appropriate concentration for each screening step as follows: 200-fold for the second screening step, 10-fold for the third screening step, and 2000-fold for the fourth screening step. Following this, 10 μL of each of the diluted mixtures was spotted onto a solid medium plate containing the appropriate antibiotics as follows: Cam + Kan for the second and third screening steps, and Amp + Kan for the fourth screening step. Each screening step was performed in triplicate.

For the fifth and sixth screening steps, the donor and recipient cells were collected by centrifugation, and each bacterial pellet was re-suspended in TNB at an OD 660 nm value of 1.8. The protocol used for the second to fourth screenings steps was then followed with some modifications as follows: the dilution ratio was optimized for each mutant, and 10 μL of each diluted suspension was streaked onto the appropriate selection plate, Tet + Kan for the fifth screening step, and Cam + Kan for the sixth screening step.

For the second to sixth screening steps, transconjugants were detected by incubating for 18–22 h at 28°C, and colony numbers were determined by either the naked eye or using a stereomicroscope. At the sixth screening step, for slower growing mutants, an additional incubation was performed either at 37°C for up to 6 h, or at 28°C overnight. In addition, the Δ*rnt*, Δ*priA*, and Δ*dnaT* mutants were incubated for 24 h at 37°C at the fifth screening step, and the presence or absence of colonies was confirmed.

### Data Analysis

For the initial screening step, the relative turbidity value of each transconjugant culture [defined as the transconjugant growth value (TGV)] divided by the median TGV value (MTGV) in the conjugation experiment set was calculated. The log_2_ value of the relative TGV (RTGV = TGV/MTGV) was defined as an arbitrary unit and calculated.

For the second to fourth screening steps, the number of transconjugant colonies for each mutant was divided by the relative turbidity value of the corresponding input recipient culture and defined as the transconjugant colony value (TCV). Then, by using the control strain BW25113 carrying pBBR122Δ*Cm*^R^ shown in Table 1, the average TCV (ATCV) of seven control reactions in each conjugation experiment set (defined as the ATCV_ctrl_) was calculated. By summarizing the results of the triplicate experiments, the log_2_ value of the relative TCV [RTCV = Average (TCV1, TCV2, and TCV3)/Average (ATCV_ctrl_1, ATCV_ctrl_2, and ATCV_ctrl_3)] was defined as an arbitrary unit and calculated.

For the fifth and sixth screening steps, the absolute value of the conjugation efficiency (transconjugants/output recipient) was calculated for each mutant.

Statistical analyses were performed using either Microsoft Excel (version 16.21) or the public domain R program (version 3.3.3).

## Results

### Refinement and Characterization of the Recipient Mutants for IncP1α Conjugation

**FIGURE 1:**
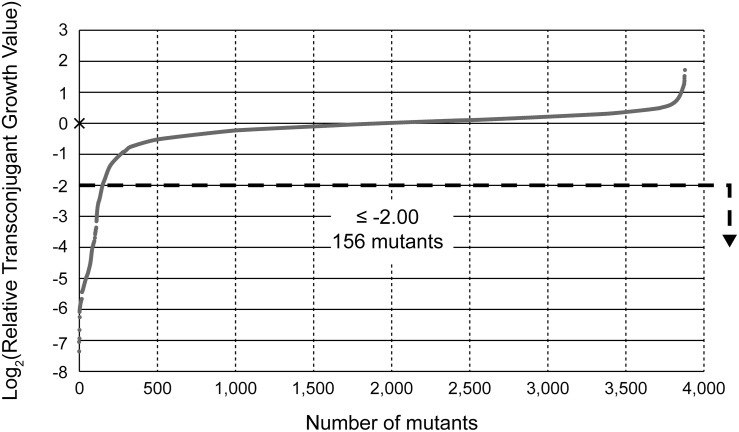
Distribution pattern of the conjugation efficiency of Keio mutants at the primary screening step. Log_2_ values of each relative transconjugant growth value (RTGV) for 3884 mutants are plotted in ascending order. For one mutant (Δ*holC*), the log_2_(RTGV) could not be calculated since transconjugant growth was less than the detectable limit, and the “×” symbol shows this. Mutants with a log_2_(RTGV) ≤−2 were subjected to the next round of screening. HB101 (pRH220 pRS316:*oriT*^*P*^) was used as the donor.

An overall flowchart of the screening is shown in Supplementary Figure S1. In the initial screening step, we detected transconjugants that had accepted both the plasmids, namely pRS316:*oriT*^*P*^ and pRH220. The growth of most transconjugants, which arose from the mutant strains, reached saturation. As shown in Figure 1, the distribution pattern of the log_2_(RTGV) values deviated from a Gaussian distribution with a tail skewed to lower values (average = −0.193, median = 0.000), but was closer to a Gaussian distribution when the samples that had a log_2_ value greater than −2, were selected (average = −0.030, median = 0.012). Since the latter distribution was expected to represent the distribution of non-conjugation-deficient mutants, 156 mutant strains, which showed log_2_ values equal to or less than −2 (i.e., one-fourth of the median value), were selected for analysis in the second screening step. Among them, Δ*priA* mutant was included, since the log_2_ value of this mutant was −5.06 (i.e., a 0.030-fold change compared to the median value). A deficiency in *priA* causes a deficiency in replication by ColE1-like *ori* (Lee and Kornberg, 1991), and pRS316:*oriT*^*P*^ was replicated using an identical origin of replication (Table 1). Therefore, we determined that this would be a useful indicator of the success of this screening system, since recipient chromosomal factors, which are critical for the acceptance of RP4, have not been identified.

The method used in the initial screening step measured the cell growth of the transconjugants included in each conjugation reaction mixture. A different method was required to select the mutant strains that showed low conjugation efficiency as well as those that exhibited a slow growth phenotype. As a result, 156 candidates were further screened by counting the number of transconjugant colonies based on the transfer of pRH220. Twenty-nine mutant strains, which showed log_2_(RTCV) values of less than −2 (i.e., one-fourth of the control), were identified by this method. These 29 mutants, in which the reason for the relatively low conjugation efficiency was not clear, were termed the “down” mutant pool, and third and fourth screenings were performed to determine the reason for this dip in conjugation efficiency (Table 2).

**TABLE 2:** Characteristics of conjugation-deficient recipient mutant pool.

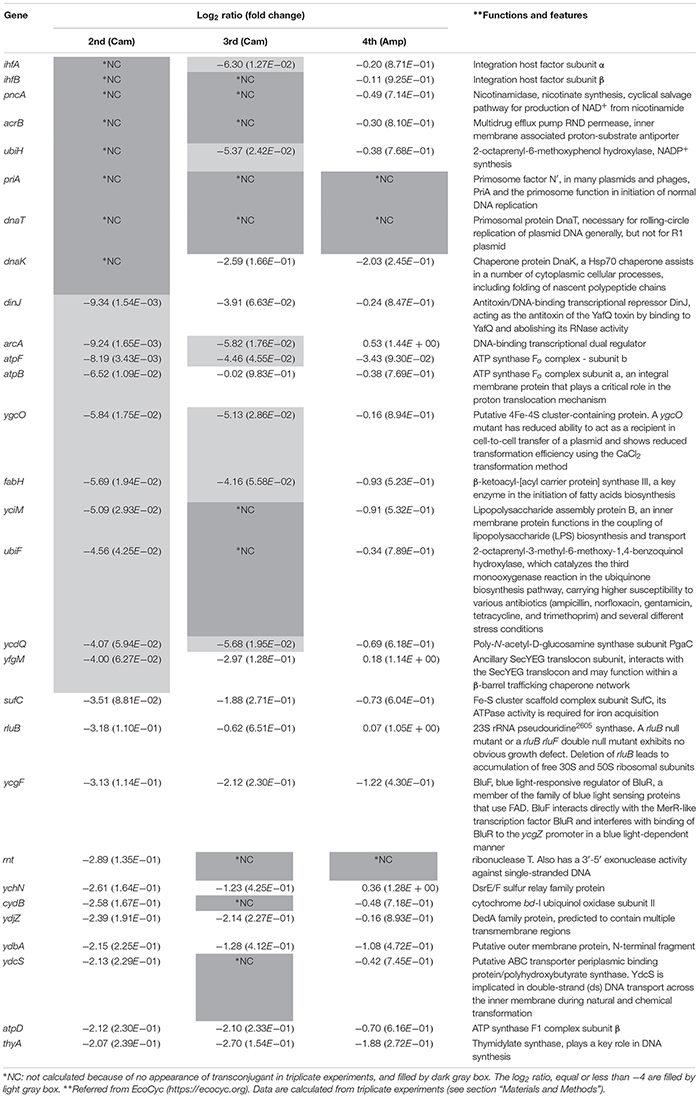

To characterize this mutant pool further, we tried to select factors affecting the transfer from donor to recipient (the third screening step). For this, a donor strain, S17-1 λ*pir* [pJP5603Δ*Km*^R^(::*Gm^R^ Cm^R^ leuB*-*D*)], in which RP4 was integrated into the chromosome, was used. The plasmid in this strain has an RP4 *oriT* and an R6K *ori*, and its replication is *pir-*dependent. As a result, the transferred plasmid is non-replicable in the recipient Keio mutants, and so transconjugants, arising as a result of homologous recombination at the *leuB*-*D* locus on the recipient chromosome, could be detected. In this assay system, mutants related to plasmid replication were expected to show normal conjugation efficiency, whereas mutants related to the transfer from donor to recipient were expected to show low conjugation efficiency. As a result, 17 mutant strains were isolated as low conjugation efficiency mutants [log_2_(RTCV) values < −4; Table 2].

In the third screening step, not only mutants related to transfer but also those related to the establishment of Cam resistance were included. To distinguish these two phenotypic differences, we used HB101 [RP4Δ*Km*^R^(::*Gm*^R^)] as the donor strain and assessed for conjugation efficiency using Amp (fourth screening step). As a result, three mutant strains, namely Δ*rnt*, Δ*priA*, and Δ*dnaT*, were isolated as possible mutants affecting transfer from donors to recipients [log_2_(RTCV) values < −4; Table 2].

### Absence of Recipient Mutants Affecting the Transfer

Interestingly, the functions of the deleted genes in the three mutants were ribonuclease (*rnt*) and DNA replication (*priA* and *dnaT*). These functions appear to be unrelated to the donor to recipient transfer. We observed that the appearance of transconjugant colonies in the Δ*atpF* mutant was delayed and that the Δ*rnt* mutant formed extremely small colonies in one of the triplicate experiments performed during the second screening step, suggesting that the conjugation deficiency of these three mutants might be superficially derived from the extremely slow growth phenotype. In addition, the analyses described above were based on relative conjugation efficiencies calculated compared to a control. Thus, to finally identify true conjugation mutants, the absolute conjugation efficiency values were measured using a more sensitive technique (fifth screening step). It is noteworthy that in the fourth screening step, detection of RP4Δ*Km*^R^(::*Gm*^R^) transfer was assessed using Amp. However, to further eliminate the possibility that the low conjugation efficiency in the three mutants was dependent on Cam or Amp, we used Tet instead, and assessed whether the low conjugation efficiency of these three mutants was superficial and caused by the extremely slow growth of the transconjugants.

**FIGURE 2:**
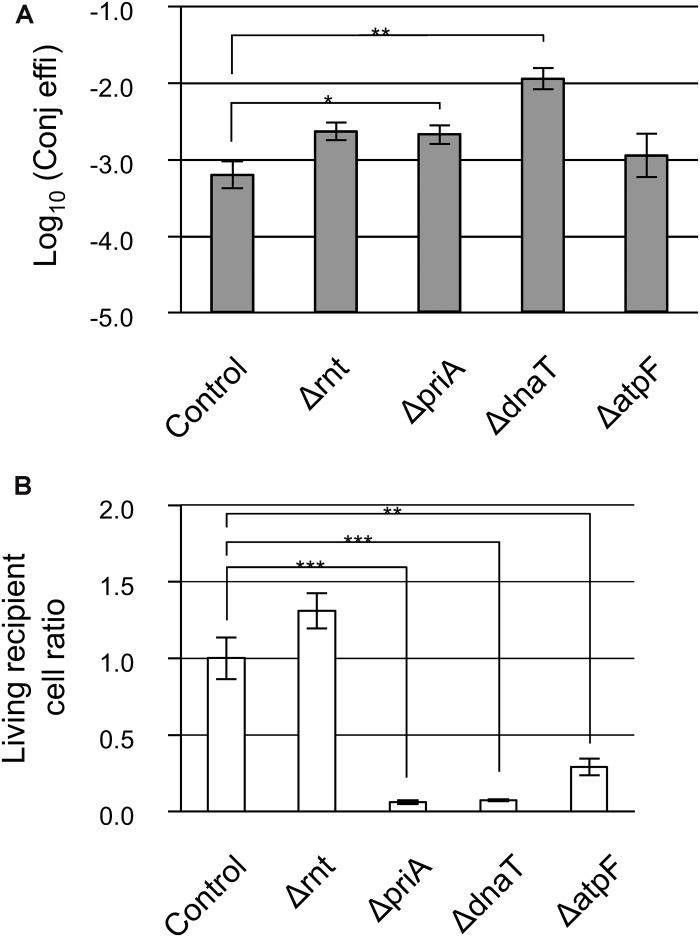
Confirmation analysis of the low conjugation efficiency in Δ*rnt*, Δ*priA*, and Δ*dnaT* mutants. **(A)** Conjugation efficiency of the candidate mutants. Bars represent the log_10_ converted values of the conjugation efficiency (transconjugants/recipient cell) and are shown as “log_10_(Conj effi).” **(B)** Relative recipient cell ratio of mutants in the conjugation reaction. Data are presented as mean ± standard error (SE). Asterisks indicate statistically significant differences: ^∗^*p* < 0.05, ^∗∗^*p* < 0.01, and ^∗∗∗^*p* < 0.001 (two-tailed *t*-test). Conjugation experiments for each mutant were performed at least thrice. HB101 (RP4Δ*Km^R^::Gm^R^*) was used as the donor, and BW25113 (pBBR122Δ*Cm*^R^) was used as the control.

The transconjugants were visualized after incubation for 24 h at 37°C using a stereomicroscope, and for 48 h at 37°C by the naked eye, all three mutants showed no reduction in conjugation efficiency (Figure 2A). Thus, these data strongly suggest that the low conjugation efficiency observed was indeed superficial and was mainly caused by the extremely slow growth of the transconjugants. Interestingly, despite the fact that the turbidity was adjusted among the recipient cell suspensions, the Δ*priA* and Δ*dnaT* mutants showed a lower number of recipient colonies, approximately 6 and 7% of the control strains, respectively (Figure 2B). Therefore, the living cell (capable of proliferation) ratio should be low in these two mutant strains.

Taken together, we conclude that there is no apparent defective recipient mutant for IncP1α conjugal transfer in these Keio mutants, and even if one existed, it appears that it would only have a mild phenotype.

### Inhibition of the Establishment of Antibiotic Resistance in a Recipient Cell Could Be an Important Step in Blocking the Spread of Antibiotic Resistance Genes

Next, we looked for the existence of mutants that showed low conjugation efficiency as a result of the inhibition of Cam resistance induced by the transferred resistance gene. Except for Δ*priA* and Δ*dnaT*, mutants that formed no transconjugant colonies in the second screening step were selected. These were Δ*ihfA*, Δ*ihfB*, Δ*pncA*, Δ*acrB*, Δ*ubiH*, and Δ*dnaK* (Table 2).

**FIGURE 3:**
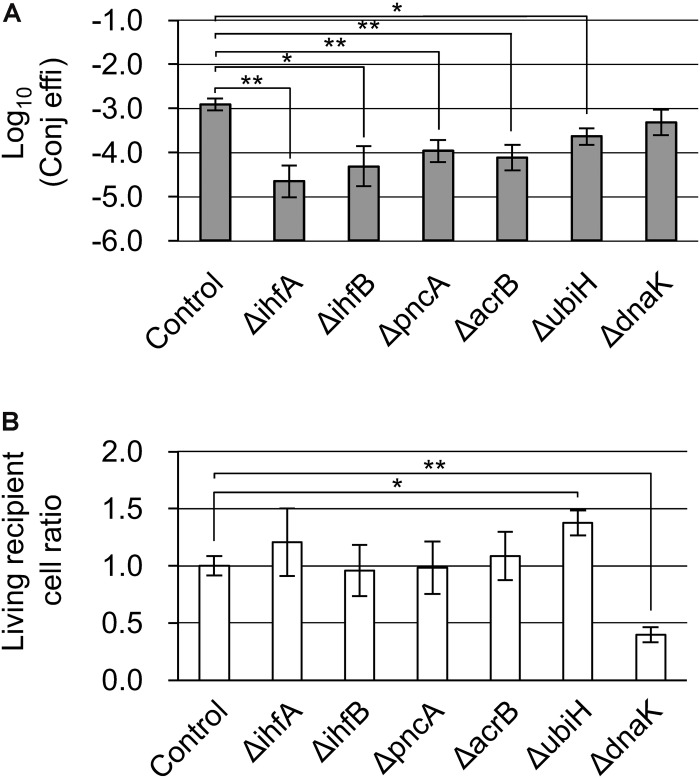
Confirmation analysis of Cam-dependent conjugation deficiency in the Δ*ihfA*, Δ*ihfB*, Δ*pncA*, Δ*acrB*, Δ*ubiH*, and Δ*dnaK* mutants. **(A)** Conjugation efficiency of the candidate mutants. Bars represent the log_10_ converted value of the conjugation efficiency (transconjugants/recipient cell) and are shown as “log_10_(Conj effi).” **(B)** Relative recipient cell ratio of the mutants in conjugation reaction. Data are presented as mean ± SE. Asterisks indicate statistically significant differences: ^∗^*p* < 0.05, ^∗∗^*p* < 0.01 (two-tailed *t*-test). Conjugation experiments in each mutant were performed in quadruplicate. HB101 (pRH220) was used as the donor, and BW25113 (pBBR122Δ*Cm*^R^) was used as the control.

With the exception of Δ*ubiH*, although the six mutants grew almost normally, their transconjugants tended to show a delay in colony formation. Furthermore, the Δ*ubiH* mutant showed delayed growth both in the recipient and in the transconjugants. Therefore, the incubation period was extended longer than that used during the second and third screenings. Except for the Δ*dnaK* mutant, an approximately 5–50-fold decrease in conjugation efficiency was observed in these mutants. The Δ*dnaK* mutant instead showed a lower living cell ratio that was approximately 40% that of the control strain (Figures 3A,B). Among these, while the conjugation efficiency of the control strain was 1.24 × 10^–3^ transconjugants/recipient, Δ*ihfA* showed the lowest conjugation efficiency (2.24 × 10^–5^ transconjugants/recipient) and a severe delay in colony formation.

## Discussion

This study examined the methods to identify effective drug targets capable of blocking antibiotic resistance arising from the conjugal transfer. While no mutants defective in transfer, including mutants defective in LPS synthesis isolated in the IncW plasmid analysis were identified, mutants with low viability and capability of establishing resistance against Cam, which was used for transconjugant selection, were isolated.

One important finding is that mutations in recipient factors that enhance plasmid transfer, such as the receptor for the RP4-pilus and factors related to the synthesis of cell surface components, which aid the interaction between donor and recipient cells, were not isolated. At the beginning of this study, we expected that we would isolate the latter factors, although they only have a moderate effect, as reported in a study using the IncW conjugal transfer system (factors for LPS synthesis; Pérez-Mendoza and de la Cruz, 2009). This is because RP4 is a plasmid with a broad host range and is expected to recognize a common structure among gram-negative bacteria. However, only the Δ*yciM* mutant, which lacked the gene for LPS assembly protein B, remained in the “down” mutant pool. This showed a severe conjugation deficiency in the third screening step (Cam selection) but was quite limited in the fourth screening (Amp selection). Therefore, the LPS assembly protein is not likely to be involved in the plasmid transfer step or the previous steps. We further attempted to reconfirm the conjugation efficiency of the Δ*rfaC* and Δ*hns* mutants. The *rfaC* gene is an LPS biosynthetic gene, and Δ*rfaC* mutant has been reported to show the strongest conjugation deficiency among all the Keio mutants in an IncW plasmid analysis (Pérez-Mendoza and de la Cruz, 2009), while *hns* was reported to function as a silencer of horizontally acquired genes (Lucchini et al., 2006; Navarre, 2006). As we expected, neither significant increase nor decrease in conjugation efficiency was observed in our IncP1α plasmid transfer analysis (Supplementary Figure S2). Thus, the IncP1α conjugal transfer system recognizes cell surface components differently from that of IncW, and the related gene(s) are probably essential genes whose knockout mutants are not included in Keio collection. As IncP1α type IV secretion system (T4SS) can transfer even to eukaryotic cells, highly conserved and essential surface structure(s) among organisms might be recognized by IncP1α T4SS. In addition, IncP1α and IncW (as Δ*hns* was not isolated from the IncW study) plasmids are adapted to escape from silencing by H-NS. All these results suggest that *E. coli* cannot escape from being a recipient organism for IncP1α plasmid transfer, and that furthermore *E. coli* does not possess any positive mechanism for incorporating genetic information by IncP1α plasmid transfer. Therefore, it is impractical to develop drugs that inhibit the transfer step in conjugation by blocking a biological process in the recipient cell.

The Δ*rnt*, Δ*priA*, and Δ*dnaT* mutants were isolated in our screening process. Theoretically, these mutants could be isolated from both the conjugation and transformation mutant screens, as they showed a severe growth deficiency. In fact, Δ*rnt* and Δ*priA* mutants were isolated in a genome-wide screening of the cell-to-cell transfer of non-conjugative plasmids (Matsuda et al., 2012). Drugs that inhibit the products of the *rnt*, *priA*, and *dnaT* genes may, therefore, be potential new antibiotics, although they will not repress conjugation.

When Cam was used for the selection of transconjugants, the Δ*ihfA*, Δ*ihfB*, Δ*pncA*, Δ*acrB*, and Δ*ubiH* mutants showed decreased conjugation efficiency as well as deficiencies for the transconjugants (Table 2, Figure 3, and Supplementary Figure S3). Based on a previous report by Liu et al. (2010), we focused on 46 Cam-sensitive Keio mutants in advance; however, only the Δ*acrB* mutant among the five conjugation-deficient mutants and only four mutants in our basic conjugation mutant pool (29 mutants), namely Δ*acrB*, Δ*cydB*, Δ*yciM*, and Δ*ygcO*, were included in the 46 mutants (Liu et al., 2010; Table 2). These results indicated that the majority of Cam-dependent conjugation-deficient mutants were not related to Cam susceptibility in the recipient cells, although such susceptible mutants were significantly enriched in the mutant pool [*p* = 0.0006 (two-tailed), Fisher’s exact test]. Therefore, although AcrB is known to be a multidrug efflux exporter (Zwama et al., 2018), the majority of genes isolated in our screening method under Cam selection probably function in the establishment of Cam resistance by the *cat* gene. Developing inhibitory drugs for the function of *ihfA*, *ihfB*, *pncA*, and *acrB* could potentially not only block the spread of the Cam resistance gene but also maintain the effect of Cam even in the presence of the resistance gene as the transconjugants derived from their knockout mutants tended to show a delay in colony formation. Such drugs could also block the spread of antibiotic resistance mediated by bacterial transduction and transformation. Future studies assessing the applicability of this type of protection method toward other antibiotics, such as fluoroquinolones, carbapenems, aminoglycosides, cephalosporins, and β-lactams with or without β-lactamase inhibitors, are anticipated. Currently, gram-negative *Enterobacteriaceae* with resistance to colistin, which is conferred by the plasmid-encoded *mcr-1* gene, is arising as a threat to human health. Moreover, recent reports have shown the presence of *mcr-1* gene in IncP plasmids (Lu et al., 2017; Saavedra et al., 2017; Zhao et al., 2017), adding to the broad transfer range and broad host range of IncP plasmids among gram-negative bacteria, poses a great threat. Hence, blocking the spread of these *Enterobacteriaceae* is urgently necessary. The data from this study might help to address this serious issue. In addition, the IncP1α transfer-based screening system developed in the present study could be applied for isolation of target genes relating to the establishment of resistance to other antibiotics.

In summary, the six-step genome-wide screening of *E. coli* recipient factors involved in IncP1α plasmid transfer revealed that blocking the function of an antibiotic resistance gene is a more practical approach than blocking conjugal transfer by targeting recipient factors to prevent the spread of antibiotic resistance genes.

## Data Availability Statement

The datasets generated for this study are available on request to the corresponding author.

## Author Contributions

KM, SY, and KS conceived and designed the experiments. KM performed the experiments. KM analyzed the data. KM, FZ, and KK contributed the reagents and materials. KM and MA wrote the manuscript. KM, FZ, MA, SY, and KK revised the manuscript. All authors read and approved the manuscript.

## Conflict of Interest

The authors declare that the research was conducted in the absence of any commercial or financial relationships that could be construed as a potential conflict of interest.
