# Supplementary material for: Targeting Antibiotic Resistance Genes Is a Better Approach to Block Acquisition of Antibiotic Resistance Than Blocking Conjugal Transfer by Recipient Cells: A Genome-Wide Screening in Escherichia coli
Source: Front Microbiol. 2020 Jan 8;10:2939. doi: 10.3389/fmicb.2019.02939 (PMC6960129; doi:10.3389/fmicb.2019.02939)
Supplement: Supplementary file 1 [file Data_Sheet_1.docx]

Supplementary Material

# Supplementary Figures


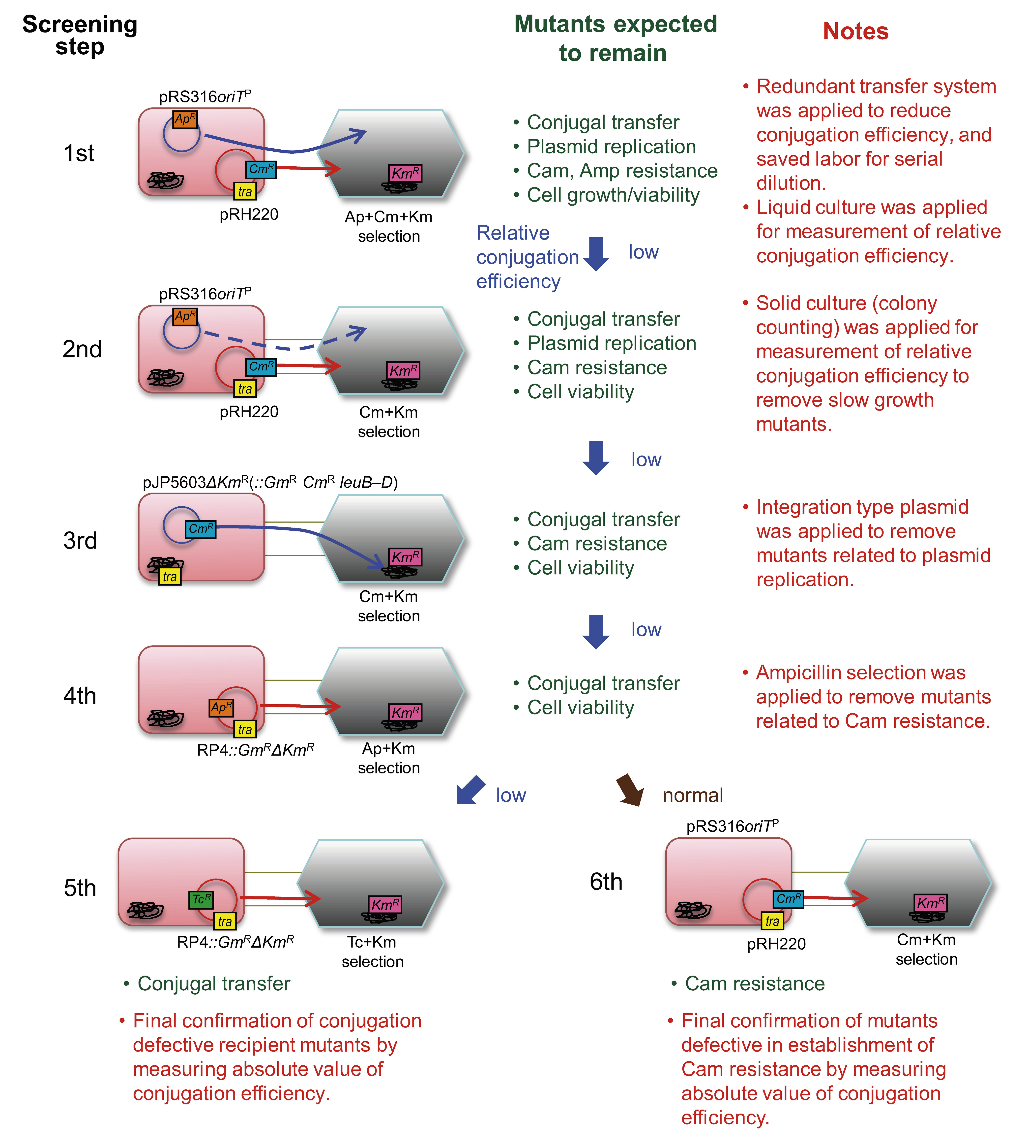


**Supplementary Figure S1. Overall flowchart of the genome-wide screening.**


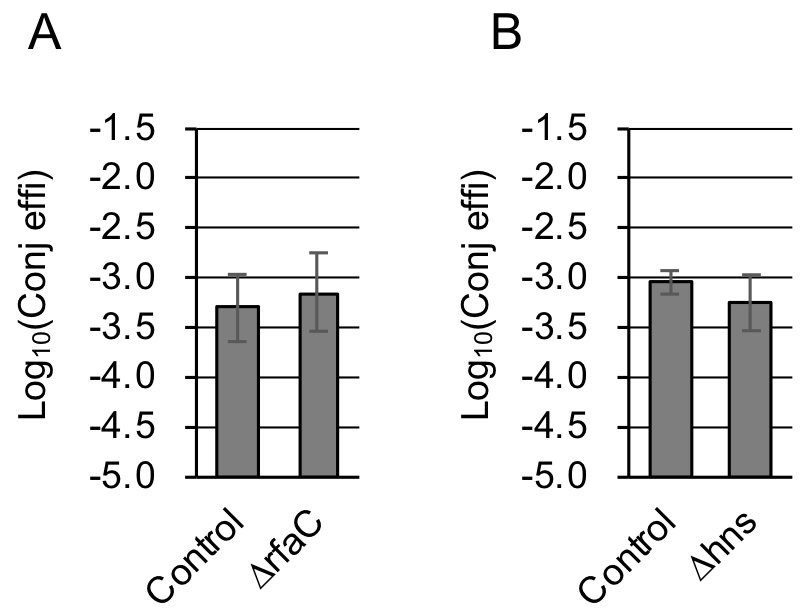


**Supplementary Figure S2.** **Confirmation analysis of normal conjugation efficiency in known defective mutants in IncW plasmid transfer (*ΔrfaC,* A) and in silencing of horizontally acquired genes (*Δhns,* B).** Bars represent the log_10_ converted values of the conjugation efficiency (transconjugants/recipient cell) and are shown as ‘log_10_(Conj effi)’. Data are presented as mean ± SE. No statistically significant differences were detected by a *t*-test (two-tailed). Conjugation experiments in each mutant were performed at least three times. HB101 (RP4*ΔKm^R^::Gm^R^*) was used as the donor, and BW25113 (pBBR122*ΔCm^R^*) was used as the control.


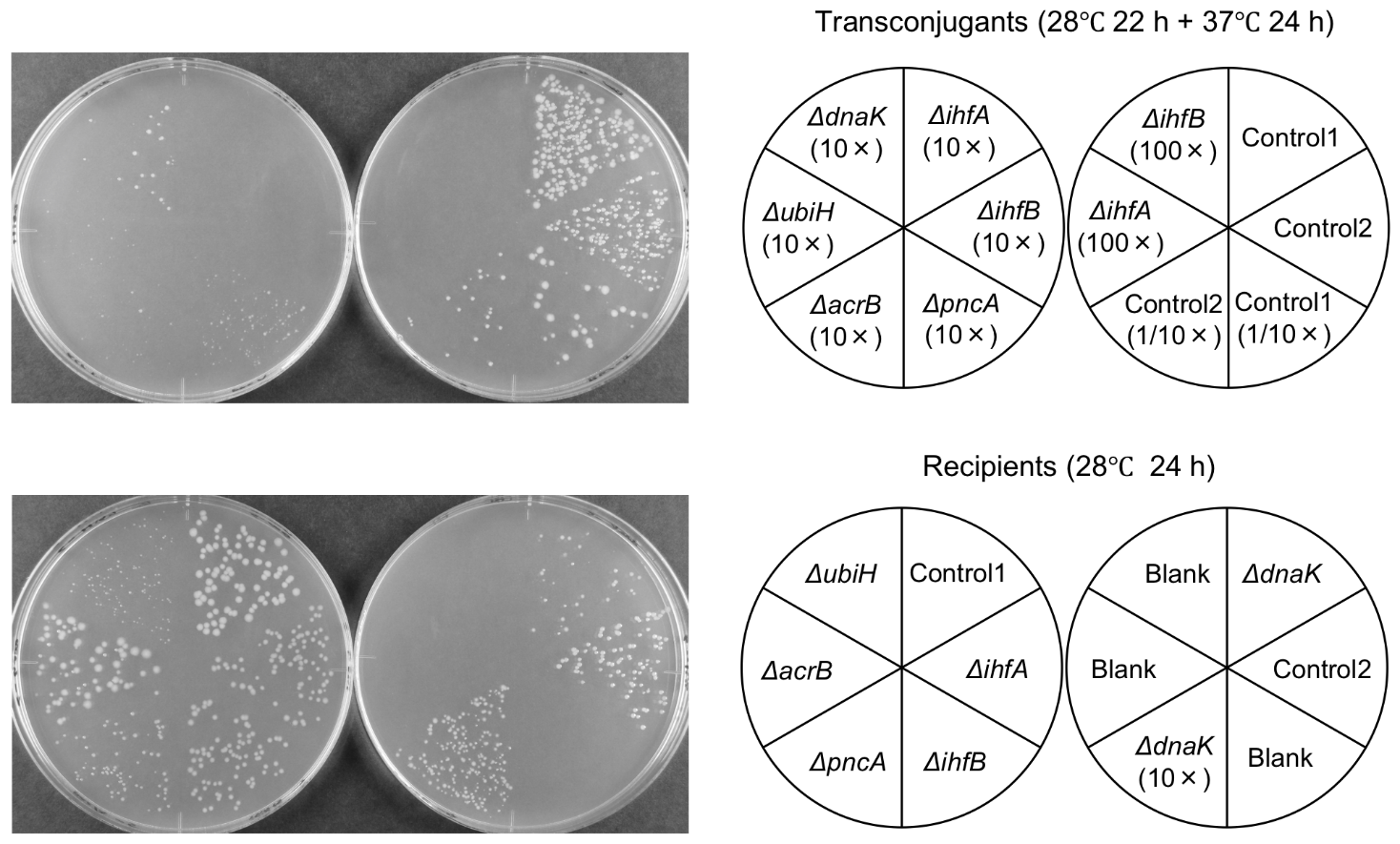


**Supplementary Figure S3.** **Growth deficiency of the *ΔihfA*, *ΔihfB*, *ΔpncA*, *ΔacrB,* and *ΔubiH* transconjugants**. Control 1 is BW25113 (pBBR122*ΔCm^R^*). Control 2 is *ΔyfeD,* a Keio mutant that does not show conjugation deficiency. The values given under each sample name represent the relative amount of reaction mixture plated against Control 1 for ease of comparison.
